# Amyloid A Amyloidosis Secondary to Thymoma

**DOI:** 10.3390/diagnostics15233040

**Published:** 2025-11-28

**Authors:** Mengyuan Li, Su Yao, Lingji Zeng, Jinghua Wang

**Affiliations:** 1 Department of Hematology, Guangdong Provincial People’s Hospital (Guangdong Academy of Medical Sciences), Southern Medical University, Guangzhou 510080, Chinazenglingji@gdph.org.cn (L.Z.); 2 School of Medicine, South China University of Technology, Guangzhou 510006, China; 3 Department of Pathology, Guangdong Provincial People’s Hospital (Guangdong Academy of Medical Sciences), Southern Medical University, Guangzhou 510080, China; 4 Guangdong Cardiovascular Institute, Guangzhou 510080, China

**Keywords:** amyloid A amyloidosis, secondary amyloidosis, thymoma

## Abstract

**Background:** Amyloid A (AA) amyloidosis is commonly secondary to chronic inflammatory diseases or malignant neoplasms. Many types of cancers have been described as inducing AA amyloidosis, usually presenting with kidney involvement. However, there are no reported cases of concurrent thymoma and AA amyloidosis. **Case Presentation:** We describe a 52-year-old male presenting chest tightness. Through a series of examinations, the patient was ultimately confirmed to have AA amyloidosis secondary to thymoma, with kidney, cardiac, nerve, and soft tissue involvement. **Conclusions:** This case represents, to our knowledge, the first reported case of systemic AA amyloidosis occurring secondary to thymoma. It highlights thymoma as a potential underlying cause of AA amyloidosis, likely due to a chronic inflammatory response driven by the tumor. This association complicates clinical management and prognosis, requiring a heightened awareness of amyloidosis in thymoma patients who present with unexplained multi-organ dysfunction.

## 1. Introduction

Amyloid A (AA) amyloidosis is characterized by the pathogenic extracellular deposition of misfolded Serum Amyloid A (SAA) protein fibrils with a low incidence of about 1–2 per million people per year [1,2]. This process is typically driven by prolonged elevated levels of SAA, an acute-phase reactant produced in response to inflammation. AA amyloidosis is most commonly secondary to chronic inflammatory diseases, such as chronic infections or autoimmune disorders. In addition, the association of AA amyloidosis with solid cancers has been described for a century, including renal cell carcinoma, lung cancer, gastrointestinal neoplasm, cutaneous cancer, and so on [3,4,5,6]. However, there are no reported cases of AA amyloidosis secondary to thymoma. Thymoma represents a notably rare neoplasm, often linked to various paraneoplastic autoimmune phenomena that may contribute to a chronic inflammatory state. While thymoma is well-recognized for its association with autoimmune conditions, such as myasthenia gravis (MG) and pure red cell aplasia, its potential role in inducing systemic AA amyloidosis has not been explored or reported deeply.

AA amyloidosis secondary to cancers usually affects organs systemically, and the kidney is the most commonly involved organ, presenting with renal dysfunction [7,8]. Meanwhile, kidney-related complications are the leading causes of death in patients with systemic AA amyloidosis [9]. Besides this, AA amyloid deposition has also been found in the digestive tract, spleen, adrenal glands, liver, etc. [10]. However, unlike other types of systemic amyloidosis, cardiac and nerve involvement are rare in AA amyloidosis [7]. Below we reported an unusual case of a patient diagnosed with thymoma and secondary AA amyloidosis with kidney, cardiac, nerve, and soft tissue involvement. We also discussed the possible pathogenic mechanisms underlying the development of AA amyloidosis secondary to thymoma. To our knowledge, this represents the first documented case of systemic AA amyloidosis induced by thymoma, highlighting both the diagnostic challenges and the importance of considering this rare association in patients with thymoma presenting with multiple organ dysfunction.

## 2. Case Report

### 2.1. Clinical Findings

**Figure 1:**
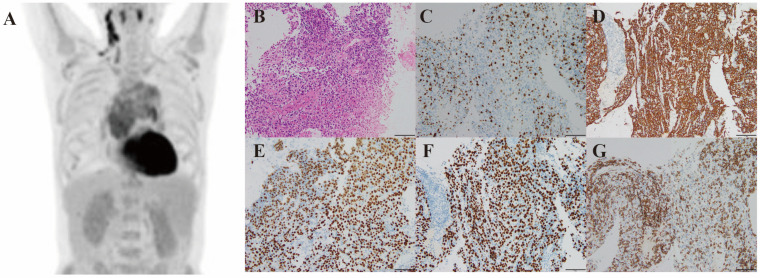
Imaging and histological features. Coronal view of the FDG PET-CT scan showing an anterior and middle mediastinal mass, multiple pulmonary lesions, and systemic lymphadenopathy (size of mass 7.0 × 12.7 cm, SUVmax 7.5) (**A**). Histopathologic appearance of mass (haematoxylin and eosin, 200×) (**B**). Immunohistochemically stained sections showing Ki-67, CK, P63, P40, CD5 expression in the mass (200×) (**C**–**G**).

A 52-year-old male patient with a one-year history of chest tightness and an unintentional 5 kg weight loss was admitted to our hospital. The clinical examination revealed multiple enlarged lymph nodes in the right neck, with a maximum diameter of 15 mm. ^18^F-deoxyglucose PET-CT showed an anterior and middle mediastinal mass with a size of 7.0 × 12.7 cm, SUVmax 5.8, multiple pulmonary lesions, and systemic lymphadenopathy (Figure 1A). The following mediastinal mass puncture revealed thymoma with amyloid deposition. At the same time, the laboratory tests revealed moderate anemia, hypoproteinemia, and renal impairment with proteinuria. The electrocardiogram showed low QRS voltages in limb leads and ST-T segment changes. The echocardiogram revealed the left ventricular wall thickened with a thickness of 15 mm, as well as decreased left ventricular diastolic function, mild mitral regurgitation, and a small amount of pericardial effusion. The left ventricular ejection fraction was 61% and E/E’ was 22. The cardiac MRI showed increased ventricular wall thickness and extracellular volume, indicating cardiac amyloidosis. Elevated levels of N-terminal probrain natriuretic peptide (NT-proBNP) and high-sensitivity cardiac Troponin T (hs-cTnT) provided additional biochemical support for cardiac involvement. The 99mTc-PYP was negative, excluding the possibility of transthyretin amyloidosis. In addition, the electromyography indicated peripheral nerve injury. Furthermore, we performed a labial salivary gland (LSG) biopsy for this patient suspected of systemic amyloidosis, and the results confirmed AA amyloidosis.

Based on these findings, the patient was eventually diagnosed with B3 thymoma and secondary AA amyloidosis involving the kidney, heart, nerve, and soft tissue. He refused surgery and chemotherapy and was discharged after receiving symptomatic treatment such as blood transfusion. After three years of follow-up, he was still alive but experienced worsening symptoms.

### 2.2. Laboratory Findings

The laboratory tests revealed moderate anemia (hemoglobin = 63 g/L), hypoproteinemia (albumin = 25.70 g/L), renal impairment (serum creatinine = 205.92 umol/L, blood urea nitrogen = 12.54 mmol/L), and proteinuria (24 h urine protein = 2360.45 mg). The levels of NT-proBNP and hs-cTnT were significantly elevated to 19,247 pg/mL and 100.9 pg/mL, respectively. The liver function was normal (alanine aminotransferase = 22 U/L, aspartate aminotransferase = 28 U/L). The carcinoembryonic antigen was increased to 0.79 ng/mL. Serum immunofixation electrophoresis and urine immunofixation electrophoresis were both negative. κfree light chain was 231.41 mg/L and λ free light chain was 242.44 mg/L, and light chain κ/λ ratio was normal. The anti SS-A (52) antibody and anti-mitochondrial M2 (AMA-M2) antibody were doubtful positives. The interleukin-6 (IL-6) was increased to 25.4 pg/mL (Table 1).

### 2.3. Histopathological Findings

The histopathological report of mediastinal mass puncture suggested B3 thymoma with amyloid deposition. Due to limited tissue, specific classification could not be performed. Immunohistochemical results of mediastinal mass: Ki-67 (30% of hot spots), CK, P63, P40, TdT, CD3, CD5, CD20, PAX5, and Lambda and Kappa were positive, while CK7, CK20, EMA, TTF1, NUT, CD117 and EBERs-ISH were negative (Figure 1B–G). The lymph node biopsy showed reactive hyperplasia of lymph node, accompanied by monocytoid B-cell hyperplasia.

**Figure 2:**
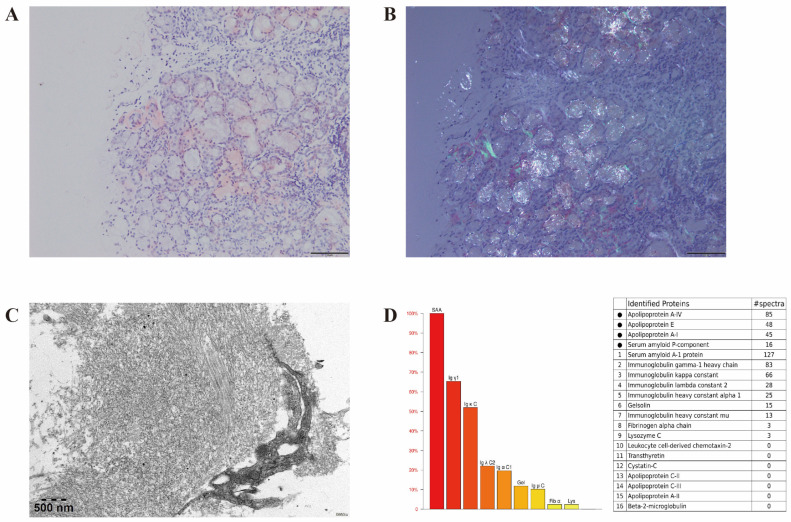
Histopathologic appearance of labial salivary gland amyloidosis. Amyloid deposits are positive for Congo red stain (**A**) and show apple green birefringence under polarized light (**B**). Electron microscopy shows amyloid fibrils (**C**). Mass spectrometry identifies high abundance of amyloid chaperone proteins including ApoA-IV and ApoE, as well as amyloidogenic protein serum amyloid A within the deposits (**D**).

The LSG biopsy showed positive Congo red staining and apple green birefringence under polarized light microscopy. Characteristic amyloid fibrils were detected by electron microscopy (EM) and identified as AA subtype through laser microdissection and mass spectrometry (MS) (Figure 2).

The bone marrow biopsy showed that bone marrow proliferation was active; there were a large number of proliferate plasma cells, the ratio of granulocytes to erythrocytes was normal, and no abnormalities were found in megakaryocytes. Immunohistochemical results of bone marrow: CD138 (plasma cells +), MUM1 (plasma cells +), Kappa (plasma cells +++), Lambda (plasma cells +), CD19 (+++), CD56 (partial +), and CD117 (−). The Congo red and oxidized Congo red were both negative. As a result, there was no evidence of clonal plasma cell proliferation.

## 3. Discussion

We present a rare case of AA amyloidosis secondary to thymoma, with multiple organ and tissue involvement. This association is infrequent, as thymomas are more typically associated with autoimmune diseases rather than amyloid deposition [11]. The diagnosis was crucial and was definitively established by combining histopathology with EM and MS, which precisely identified the AA subtype. We report this case and discuss the mechanism between AA amyloidosis and thymoma.

The discovery of AA amyloidosis in conjunction with malignant neoplasms has been reported for many years, in localized or systemic forms. Renal cell carcinoma is the most frequent type of solid malignance associated with systemic AA amyloidosis, accounting for up to one-quarter to nearly half of all such reported cases [12,13]. In contrast, localized AA amyloidosis is a rare phenomenon. It was predominantly observed in endocrine tumors, such as medullary carcinoma of the thyroid and pancreatic insulinoma, where the amyloid fibrils may result from tumor apoptosis [14,15]. Additionally, other types of solid cancers have been reported to induce AA amyloidosis. In 2020, Fujisawa et al. reported a case of systemic AA amyloidosis secondary to cervical cancer, affecting both the kidney and gastrointestinal tract. The patient underwent an extended hysterectomy and chemotherapy; however, the amyloidosis recurred after cytomegalovirus infection and cancer relapse, leading to end-stage renal disease and death [16]. In 2013, Gueutin et al. reported a case of renal AA amyloidosis associated with non-small cell lung cancer presenting as nephrotic syndrome. The renal function continued to deteriorate and the patient received palliative care [3]. The list of different solid cancers associated with systemic AA amyloidosis reported is shown in Table 2 [3,4,5,6,16,17,18,19,20,21,22,23,24,25,26,27]. However, there have been no reports on the co-existence of AA amyloidosis and thymoma, apart from a few cases of localized thymic amyloidosis. In 2004, Takamori et al. reported a case of localized thymic AA amyloidosis. The patient recovered well after the removal of the amyloid tumor and bilateral thymectomy [28]. In 2010, Kato et al. reported a case of sclerosing thymoma-like thymic amyloidoma with nephrotic syndrome [29]. Overall, only eight cases of thymic amyloidosis have been reported, and one patient was diagnosed with AA amyloidosis, while the others were all diagnosed with amyloid light chain amyloidosis [30]. To our knowledge, this is the first case of systemic AA amyloidosis induced by thymoma.

In this case, the patient was first diagnosed with thymoma and amyloid deposition through histopathological examination. Considering the patient’s symptoms of heart failure and renal failure, as well as the results of the echocardiogram and cardiac MRI indicating cardiac amyloidosis, it suggested that this patient might have systemic amyloidosis. We first ruled out the possibility of transthyretin amyloidosis and light chain amyloidosis through 99mTc-PYP and bone marrow biopsy, respectively. Then further histopathological examination was needed to confirm the presence of amyloidosis. Due to the patient’s severe condition and difficulty in tolerating organ biopsy, we performed superficial tissue LSG biopsy, which had been demonstrated to be safer [31].

Identification of the specific type of amyloidosis is crucial for guiding treatment. Nevertheless, traditional methods like immunohistochemistry (IHC) and immunofluorescence (IF) have some limitations. IHC results depend highly on staining of amyloid deposits, tissue processing, and operator expertise, which may lead to false negative or non-specific staining [32,33]. IF is more sensitive and reliable than IHC for detecting this type of amyloid deposits. However, IF requires fresh frozen tissue, which limits its practicality in routine clinical settings [33]. To overcome these diagnostic challenges, immunoelectron microscopy (IEM) and mass spectrometry (MS) offer superior accuracy and reliability in amyloid identification and typing. IEM combines IHC and EM to visualize amyloid fibrils at the ultrastructural level. It uses gold-labeled secondary antibodies to colocalize the protein within amyloid fibrils, significantly reducing background staining and overcoming the limitations of non-specific staining inherent in conventional IHC [34]. MS enables the simultaneous identification of all protein constituents within the amyloid deposits, including the major fibril protein and a range of associated chaperone proteins such as Apolipoprotein E, Apolipoprotein A-IV, and Serum Amyloid P-component [35]. Detecting these chaperone proteins provides concrete molecular evidence supporting the diagnosis of amyloidosis. In recent years, MS has been considered a new diagnostic standard for amyloid typing. In our case, through pathological Congo red staining, EM, and MS examination, the patient was ultimately confirmed to had AA amyloidosis.

AA amyloidosis secondary to thymoma may be induced by a cascade of events originating from a loss of immune tolerance. The normal thymus is essential for T-cell maturation and the establishment of central immune tolerance. However, compared to the normal thymus, the expression of autoimmune regulators significantly decreases in thymoma, which is essential for negative selection and elimination of self-reactive T cells [36]. Additionally, in thymoma, decreased MHC class II expression compromises self-antigen presentation and tolerance induction, consisting of an abnormal thymus microenvironment. As a result, while some T cells could complete positive selection, they failed to complete negative selection, allowing self-reactive T cells to escape central tolerance [37]. The export of such autoantigen-specific T cells may disturb circulating T-cell subset composition, disrupt peripheral immune homeostasis, and directly or indirectly initiate immune attacks against self-tissues, thereby significantly increasing the risk of autoimmune diseases [38]. MG is the most common autoimmune disease among thymoma patients, followed by systemic lupus erythematosus and pure red cell aplasia [39,40,41]. As we know, autoimmune diseases are the main cause of secondary AA amyloidosis, with the core mechanism revolving around chronic inflammation driving the sustained overproduction of SAA [34]. This is exemplified by conditions such as rheumatoid arthritis, ankylosing spondylitis, and Crohn’s disease, all of which are characterized by prolonged periods of inflammation. The persistent inflammatory state results in the continuous expression of pro-inflammatory cytokines, particularly IL-6, as well as tumor necrosis factor (TNF) and IL-1. These cytokines dramatically stimulate the liver to upregulate the production of SAA, an acute-phase reactant. Under normal conditions, the plasma concentration of SAA is low; however, during a chronic acute-phase reaction, it can increase by as much as 1000-fold [10,42]. In our case, the anti SS-A (52) antibody and AMA-M2 antibody were doubtful positives, perhaps indicating the existence of an autoimmune response.

Additionally, association of cancer and AA amyloidosis seems to be mediated by the malignant cells and the tumor microenvironment. For example, the malignant cells can directly produce SAA, or indirectly secrete pro-inflammatory cytokines such as IL-1, IL-6, and TNF-α, which can signal the liver to produce SAA [43,44,45]. There have been some case reports of IL-6-producing thymic carcinoma, including squamous cell carcinoma and undifferentiated thymic carcinoma [44,46]. Interesting, the IL-6 was increased to 25.4 pg/mL in our case. In addition, anti-tumor lymphocytes or macrophages can also produce pro-inflammatory cytokines to induce endogenous production of SAA. Furthermore, inflammatory cytokines have been shown to inhibit monocyte-mediated SAA degradation in vitro, leading to excess AA amyloid deposition [14].

Furthermore, cancer therapy, such as checkpoint inhibitors (ICIs), has been reported in association with AA amyloidosis. The ICI can induce an increased release of cytokines in a T-cell-mediated hyperinflammatory state. Notably, significantly elevated levels of IL-6 and/or TNF-α may play a role in the onset of AA amyloidosis. In 2020, Lapman et al. reported a series of clinical cases confirming this association, documenting renal AA amyloidosis in patients receiving PD-1 inhibitors (pembrolizumab, nivolumab) for various malignancies, including colon cancer, lung cancer, and melanoma [47]. A critical observation is that this complication can arise or progress even after achieving oncological remission and discontinuing ICI therapy. This highlights the significant impact of immune dysregulation caused by the treatment itself.

According to the reports, the kidney is the most commonly involved organ, and renal dysfunction is the most common clinical manifestation in secondary AA amyloidosis. Unlike other types of systemic amyloidosis, cardiac and nerve involvement are very rare in AA amyloidosis [7]. However, in our case, the patient had widespread organ damage including kidney, heart, and nerve, and was found to be in NYHA class III. The existing study revealed that the molecular mechanism of thymoma may be related to immune inflammation, suggesting that multiple organ involvement in our case may be associated with severe inflammation caused by thymoma [48].

The treatment methods for AA amyloidosis secondary to cancer mainly include anti-tumor therapy that inhibits production of SAA and symptomatic treatment that maintains organ function. A study has shown that anti-TNF drugs are effective in treating AA amyloidosis, though they may increase the risk of infection [49]. In addition, the humanized anti-IL-6 receptor antibody tocilizumab has shown efficacy in suppressing SAA production and promoting the regression of amyloid deposits in some patients, including those refractory to anti-TNF therapy [47]. In addition, targeted therapy aiming to inhibit amyloid formation or remove misfolded protein, such as eprodisate and miridesap, has been attempted in recent years [50,51]. In our case, the patient only received symptomatic treatment due to personal reasons. He is still alive now, but his symptoms are worsening.

## 4. Conclusions

This case highlights thymoma as a cause of AA amyloidosis, as well as the need to suspect amyloid formation and deposition among patients with thymoma. Meanwhile, LSG biopsy combined with IEM and/or MS is an effective tool in the diagnosis of systemic AA amyloidosis. Early recognition and appropriate treatment are crucial for improving the prognosis of these patients.

## Figures and Tables

**Table 1:** Summary of significant laboratory tests.

Parameter	Value	Normal Range
White blood cell (×10^9^/L)	9.72	3.5–9.5
Hemoglobin (g/L)	63	130–175
Platelet (×10^9^/L)	422	125–350
C-reactive protein (mg/L)	71	0–10
Carcinoembryonic antigen (ng/mL)	0.97	0–5
Interleukin-6 (pg/mL)	25.4	0–7
Alanine aminotransferase (U/L)	22	9–50
Aspartate aminotransferase (U/L)	28	15–40
Serum total protein (g/L)	108.2	65–85
Serum albumin (g/L)	25.70	40–55
Serum creatinine (umol/L)	173.49	57–97
Blood urea nitrogen (mmol/L)	12.54	3.1–8.0
24 h urine protein (mg/24 h)	2360.45	0–150
N-terminal probrain natriuretic peptide (pg/mL)	19247	0–125
High-sensitivity cardiac Troponin T (pg/mL)	100.9	0–14
Serum immunoglobulin		
IgG (g/L)	81.34	7–16
IgA (g/L)	10.43	0.7–4
IgM (g/L)	3.72	0.4–2.3
κ-chain (g/L)	20.86	0.93–2.42
λ-chain (g/L)	12.13	1.17–2.93
Serum free light chains		
κ-chain (mg/L)	231.41	3.3–19.40
λ-chain (mg/L)	242.44	5.71–26.30
κ/λ ratio	0.9545	0.26–1.65

**Table 2:** Different solid cancers associated with systemic AA amyloidosis.

Author, Year	Cancer Type	Involved Organs	Outcome
Beck et al., 1983 [4]	Basal cell carcinoma	Kidneys, the lymph nodes, the spleen	Improvement
Thysell et al., 1986 [17]	Hepatocellular adenoma	Kidney, liver vessels	Nephrotic syndrome resolved, normal renal function
Richmond et al., 1990 [18]	Bronchial carcinoma	Kidney	Died
Miranda et al., 1994 [19]	Ovarian carcinoma	Rectal and renal	Died of postoperative renal insufficiency
Chiaramonte et al., 2002 [20]	Retroperitoneal follicular dendritic cell sarcoma	Liver	Died of metastatic tumor.
Agha et al., 2002 [21]	Pleomorphic sarcoma of the spleen	Kidney, cardiac	Proteinuria declined, clinical improvement
Kanat., 2003 [22]	Small cell carcinoma of the bladder	Rectal and renal	Died of progressive disease
Garthwaite et al., 2003 [23]	Bronchial squamous cell carcinoma	Kidney	Hemodialysis
Nobata et al., 2012 [24]	Metastatic lung tumor from RCC	Kidney	Hemodialysis
Gueutin et al., 2013 [3]	NSCLC	Kidney	Renal function worsening
Babu et al., 2014 [6]	RCC	Kidney, gastrointestinal tract, liver, spleen	Dialysis-dependent, systemic improvement
Dervisoglu et al., 2015 [25]	Paragangliomas	Kidney and rectal	Died of progressive disease
Fujisawa et al., 2021 [16]	Cervical cancer of the uterus	Kidney, gastrointestinal tract	Remission after resection, later relapse
Wang et al., 2021 [26]	Lung squamous cell carcinoma	Peritoneum, mesentery	Died of respiratory infection
Soni et al., 2023 [5]	Gastric adenocarcinoma occurred with concomitant gastric GIST	Spleen	Not applicable
Obara et al., 2024 [27]	Proliferating pilomatricoma	Gastrointestinal tract (colon, ileum, rectum)	Remission of amyloidosis

RCC, Renal cell carcinoma; NSCLS, Non-small cell lung cancer; GIST, gastrointestinal tumor.

## Data Availability

The datasets used during the current study are available from the corresponding author on reasonable request.
